# Preferences for infant delivery site among pregnant women and new mothers in Northern Karnataka, India

**DOI:** 10.1186/s12884-015-0481-8

**Published:** 2015-02-27

**Authors:** Sharon G Bruce, Andrea K Blanchard, Kaveri Gurav, Anuradha Roy, Krishnamurthy Jayanna, Haranahalli L Mohan, Banadakoppa M Ramesh, James F Blanchard, Stephen Moses, Lisa Avery

**Affiliations:** University of Manitoba, Winnipeg, Canada; Karnataka Heath Promotion Trust, Bangalore, India

**Keywords:** Delivery sites, Rural India, Quality of care, Qualitative, Reproductive health

## Abstract

**Background:**

The National Rural Health Mission (NRHM) of India aims to increase the uptake of safe and institutional delivery among rural communities to improve maternal, neonatal and child health (MNCH) outcomes. Previous studies in India have found that while there have been increasing numbers of institutional deliveries there are still considerable barriers to utilization and quality of services, particularly in rural areas, that may mitigate improvements achieved by MNCH interventions. This paper aims to explore the factors influencing preference for home, public or private hospital delivery among rural pregnant and new mothers in three northern districts of Karnataka state, South India.

**Methods:**

In-depth qualitative interviews were conducted in 2010 among 110 pregnant women, new mothers (infants born within past 3 months), their husbands and mothers-in-law. Interviews were conducted in the local language (Kannada) and then translated to English for analysis. The interviews of pregnant women and new mothers were used for analysis to ultimately develop broader themes around definitions of quality care from the perspective of service users, and the influence this had on their delivery site preferences.

**Results:**

Geographical and financial access were important barriers to accessing institutional delivery services in all districts, and among those both above and below the poverty line. Access issues of greatest concern were high costs at private institutions, continuing fees at public hospitals and the inconsistent receipt of government incentives. However, views on quality of care that shaped delivery site preferences were deeply rooted in socio-cultural expectations for comfortable, respectful and safe care that must ultimately be addressed to change negative perceptions about institutional, and particularly public hospital, care at delivery.

**Conclusions:**

In the literature, quality of care beyond access has largely been overlooked in favour of support for incentives on the demand side, and more trained doctors, facilities and equipment on the supply side. Taking a comprehensive approach to quality of care in line with cultural values and community needs is imperative for improving experiences, utilization, and ultimately maternal and neonatal health outcomes at the time of delivery.

## Background

While motherhood is a positive and fulfilling experience for many women, pregnancy and childbirth can also be associated with suffering, ill health and death. In 2000, the Millennium Development Goals (MDGs) were developed at a United Nations summit, and two of the eight MDGs relate specifically to maternal and child health: reducing child mortality (MDG 4) and improving maternal health (MDG 5). While some improvement has been made since 2000, for many areas of the world, pregnancy, childbirth and early childhood remain major contributors to morbidity and mortality. For example, globally in 2009, 3.3 million newborns died within the first week of life, and in 2010, 287,000 women died from complications that could have been prevented, including severe bleeding, infections, and eclampsia [1,2]. Life-long disabilities related to childbirth, such as obstetric fistula for women and cerebral palsy for infants, also remain major problems. Importantly, morbidity and mortality during pregnancy, childbirth and early childhood are not evenly distributed globally; 99% of maternal and neonatal morbidities and mortalities occur in low income countries, with more than half occurring in sub-Saharan Africa and one third occurring in South Asia [3].

Despite recent economic growth and success, India continues to be faced with poor maternal and child health outcomes. India accounts for more than 20% of the global burden of maternal mortality, with 187 deaths per 100 000 live births [4]. The infant mortality rate is 44 per 1000 live births and almost one-third of global neonatal deaths occur in India (neonatal mortality rate is 32 per 1000 live births) [5]. In addition, only 57% of births in India in 2009 were assisted by a skilled birth attendant [6]. While the proportion of institutional deliveries is increasing, up to nearly 70% in 2013–14 [7], the skill level of attendants at institutions is not certain. Similar to high and middle-income countries, maternal, neonatal and child health outcomes vary along social, economic and geographic lines. Thus, a key goal of the Government of India is to improve maternal and child health, with a specific focus on the rural populations who predominantly face economic disadvantage. The Government of India launched the National Rural Health Mission (NRHM) in April 2005 to improve the access of rural populations to effective primary health care, including an increased uptake of institutional delivery among rural communities.

The Karnataka Health Promotion Trust (KHPT) and the University of Manitoba (UM) have provided technical support since 2008 to the NRHM in Karnataka state, in south India. The main goals have been to improve availability, accessibility, quality, and utilization of maternal, neonatal and child health (MNCH) services for rural communities with the poorest outcomes. A qualitative study was undertaken by KHPT and UM to elucidate social and cultural knowledge and practices associated with pregnancy, delivery and care of the neonate, which has been used to inform the outreach and communication elements of their programs. The focus of this paper is on women’s preferred site for birthing their children (i.e. home vs. government hospital vs. private hospital). Delivery site is an issue of importance in India because of the adverse outcomes for women and infants associated with unattended births and births with under-skilled attendants. Understanding women’s preferences and the factors women consider important in identifying preferred sites will assist in better understanding service use patterns and ultimately may assist with increased use of skilled attendants and institutional deliveries.

## Methods

### Study site and design

The study was conducted in Karnataka, India, which in 2011 had 61 million people, with more than two-thirds living in rural areas [8]. Of the eight districts in northern Karnataka (a more economically disadvantaged part of the state) three were chosen for the study: Bellary, Bagalkot and Gulbarga. Together these districts represent the diversity that exists in the programmatic area in relation to geographical locations, socio-demographic characteristics, and maternal, neonatal and child health outcomes of interest to the study.

Informed consent forms were read to each participant by the local researchers. Literate women provided signatures on the consent forms. Women who were not literate provided a thumb impression, which is a standard practice for research in the area. Consent forms were also signed by a witness, which included family members or Accredited Social Health Activists or ASHAs (community workers who act in a health promotion capacity). Ethical approval, including the consent process described herein was obtained from St. John’s Medical College in Bangalore, India and the Human Research Ethics Board at the University of Manitoba, Canada.

### Study participants

One hundred and ten people were interviewed, including 30 pregnant women, 30 new mothers (live birth within the past three months), 25 husbands of pregnant women or new mothers, and 25 maternal or paternal grandmothers (Table 1). This report is restricted to interviews with pregnant women and new mothers.

**Table 1:** **Number and type of participants from each study district**

	**Bellary**	**Bagalkot**	**Gulbarga**	**Total**
**Pregnant women**	10	10	10	30
**New moms**	10	10	10	30
**Husbands**	8	9	8	25
**Mothers-in-Law**	8	9	8	25
**Total**	36	38	36	110

Selection of participants was guided by the principle of “maximizing variation”, which refers to inclusion of a broad range of individuals to ensure that a wide variety of experiences are documented. Purposeful sampling was employed, in which participants were selected based on district, sub-centre, and religion or ethnicity (i.e., Muslim, lower and upper caste Hindu, including scheduled tribes, and *Devadasi*, who are women who have been dedicated to a god or goddess as part of a regional socio-historical tradition that has now overlapped with commercial sex work [9]). Inclusion criteria were being a pregnant woman or a woman who had a live birth within the past three months in public or private hospitals, or at home. Participants were identified by local community health care providers, for example auxiliary nurse midwives (ANMs). Exclusion criteria were individuals not able to provide informed consent and women who were visiting the area but did not normally access services in the area. In-depth semi-structured interviews were conducted between July and September 2010.

Analysis for this paper was restricted to pregnant women and new mothers because their perceptions on the birthing experience as service-users are important to consider when developing interventions to improve services and effect health outcomes. While women’s preferences don’t always translate into reality of the birthing site, we found that women’s opinions were taken into consideration by families in the decision-making process regarding birthing site [10].

### Data collection methods

Semi-structured interviews with open-ended questions were conducted by trained local researchers fluent in *Kannada* and English, or Hindi and English. Semi-structured interviews provide enough structure to ensure comparable responses on key concepts, but also allow freedom to add questions to explore other areas during the interview [11]. One-on-one interviews were conducted between an interviewer and the participant to allow interviewees to express themselves confidentially. The interview questionnaire focused on delivery care practices, including decision-making on delivery site, and knowledge of demand generation strategies (schemes or incentives). In relation to delivery site preferences, questions focused on where the baby was or would be delivered; the reasons for that location; what was the pregnant or new mother’s preferred delivery site and why; and delivery locations in their geographic area. Field notes were recorded by the interviewers to describe the setting and any observations about the process, or non-verbal observations at the time of the interview. After pilot testing, the research team and the interviewers met to discuss the process, and address issues and concerns. Interviews were then completed, transcribed, and translated into English by local researchers.

### Analysis

Thematic analysis, informed by ethnographic methodology, was used in the analysis. The steps in our analysis included first, a line-by-line reading of all the interviews to gain a descriptive understanding of the responses to each research question. Next, we coded the information pertaining to the research questions on *where* women wanted to deliver, and *why* they preferred that site, which was often based on the experiences or perceptions that influenced their preferences. Preferences on delivery site related primarily to issues of quality, which we categorized within three main components: a) *access (financial and geographical)*, b) *cultural and social acceptability*, and c) *safety and efficacy* of delivery care (components of each category found in Table 2). These findings were compared by district and type of participants, such as caste and education level to try to account for the differences in views. Similar components have been found to constitute quality in previous research on quality of maternal health care [12,13]. We arrived at broader themes surrounding these components of quality care by considering the range of participants’ views, broader programmatic and academic literature, and in light of the implications for MNCH interventions.

**Table 2:** **Components of quality in infant delivery care according to pregnant and new mothers in North Karnataka**

**Components of quality for delivery care**	**Sub-components**
**A) Accessibility**	● Financial: ability and/or willingness to pay and receipt of incentives
	● Geographical: ambulance brought them to hospital
	● Structural: wait times, availability of staff or Accredited Social Health Activist (ASHA)/ Auxiliary Nurse Midwife (ANM) present, availability of beds
**B) Cultural and social acceptability**	● Treatment by the nurses, doctors or ASHAs (attentiveness, honesty, respect)
	● Ability to enact cultural practices associated with childbirth
	● Availability of preferred facilities and amenities for a “normal delivery”
**C) Safety and efficacy**	● Quick delivery speed
	● Low levels of pain
	● Perceptions of safety and comfort

## Results

### Socio-demographic characteristics of pregnant and new mothers in the study

The average age of pregnant women and new mothers was approximately 21 years (see Table 3). Almost half of pregnant women (47%), and 73% of new mothers were below-poverty line status; 60% of pregnant women and 40% of new mothers had no education; and 40-50% were of the lowest (scheduled) caste. Differences by district were found. The majority of women in Bagalkot were below poverty, already had children, and were without education, compared to the other districts.

**Table 3:** **Socio-demographic characteristics of the participants**

**Characteristics**	**Category**	**Pregnant women (n = 30)**	**New mothers (n = 30)**	**Total (N = 60)**
**Age**	*Mean (years)*	20.5	21.7	21.1
**Economic status**	*Below Poverty Line*	46.6%	73.3%	60%
**Caste**	*Scheduled*	39%	53.3%	41.7%
**Education level**	*None*	60%	40%	50%
**Gravida status**	*No children*	43.3%	26.7%	35%

### Components of quality care influencing delivery site preferences

#### Accessibility

An important component of quality of care found in this study as well as others was financial, geographical and structural accessibility [12,13]. For home delivery, the issue of accessibility was not raised by participants except in relation to structural aspects of whether skilled birth attendants were available and if there was adequate space for delivery. Women reported that in most cases, knowledgeable and skilled birth attendants were available at home deliveries.

Geographical and financial access to health care services and structure of institutional delivery were discussed as important factors in accessing hospital services. Geographical access was raised as a concern when women discussed availability of ambulances to get to the hospital for delivery. Some indicated that the Accredited Social Health Activists (ASHAs) (community workers who act in a health promotion capacity, creating awareness and counseling women on safe delivery, and facilitating access to health care services) arranged for transport to the hospital, while some women in Bagalkot and Gulbarga stated that the ambulance often came late and the delivery occurred at home.

Many respondents indicated that their preference was to attend a private hospital, and lack of financial means was one reason for attending a government hospital instead. However, they further related that there were costs associated with government hospitals, such as bribes and costs for medicines and equipment (e.g., needles and sutures). Families often purchased equipment prior to the delivery and brought the items with them to the hospital.

*“Earlier they [government hospitals] were providing treatment at free cost. Nowadays, they too collect money for providing treatment”.* (New mother in Gulbarga)

Receipt of government incentives was not uniform across districts; some benefits were not received despite families meeting government requirements. Two schemes were discussed most frequently: the *Janani Suraksha Yojana* (JSY) cash transfer scheme, in which the government provides cash transfers to below-poverty-line families who deliver at government hospitals; and the *Madilu* kit, which is a post-natal care package that includes blankets, clothing, soaps, and mosquito nets for women below-poverty-line or scheduled caste or tribe status [14]. Yet the extent of receipt of incentives was often perceived as limited, as one new mother expressed,

*“They gave Rs. 600 and clothes. That’s all”.* (New mother in Gulbarga)

Interestingly, some women who did receive incentives from either scheme indicated that they would prefer private or home deliveries if they received incentives at those sites.

“*They don’t give anything for the pregnant women, but the delivery cost can be free provided you produce your BPL ration card at the time of delivery in Government hospital. Then only one can avail these schemes benefits. Any one up to two deliveries will be free of cost. But if they go for third one it will not be free, that is the policy. As mine is now second one, so I can avail this benefit. As my first delivery benefits have still not reached me till now, we did pay for it, but this time it was done free of cost*”. (New mother, Bagalkot)

*“Yes, they give blankets, but for my ‘Papu’ they have not given any because they give it to those who deliver in the government hospital and not to those who deliver in the private hospital”* (New mom, Bagalkot).

There was also the widespread perception that delivering at the government hospital is for the poor and the private was for the rich, indicating uneven perceived and actual access to private versus public institutional delivery site based on socio-economic status. This was echoed by women in both Bagalkot and Gulbarga:

*“For poor people…government. For rich people… they go to private.”* (Pregnant woman in Bagalkot)

*“…these Government hospitals are meant for poor people only. If you don’t care about us then who else can take care of us.” *(New mother, Gulbarga)

In addition, women in Bellary felt that when incentives were not received by the intended recipients who were below-poverty line status, they (below-poverty line families) could not question or demand the hospital authorities to remedy the situation.

*“Hospital says, ‘it hasn’t come,’ and so we kept quiet.”* (New mom, Bellary)

Another woman from Bellary said that the government hospital would explain to her that the incentive hasn’t come and they always say,

*“Tomorrow we will give”*. (New mother, Bellary)

Further, some expressed more explicitly that they felt they would not be listened to if they brought up the issue of incentives.

“*When they say no, what else can we say? … They will say have you lost your mind, why have you come like this? …So we don’t go and ask at all… We keep quiet.”* (New mother, Bellary)

Women in all districts mentioned high cost being a barrier to delivering at private hospitals, but associated cost with improved quality.

“*In a private hospital, they will look after well for the greediness of money… If we go to government hospital, they do not care about us.”* (Pregnant woman, Gulbarga)

Regarding structural access, there was inconsistency in the contact and support that ASHAs provided, as some in Bagalkot and Gulbarga mentioned that no ASHAs came to help or bring them to the hospital. In a couple of cases there was the lack of availability of beds at the hospital as well. As a below poverty, scheduled caste woman in Gulbarga explained, she was made to sleep outside during her short visit to the local primary health centre for a complicated delivery:

*“In the house, I started having too many problems, so in the morning itself we called the vehicle to take me to the hospital [government primary health centre]… We went at around 6 o’clock and by 10 o’clock it was over. With a lot of problems the delivery happened. After that, sister [nurse/ANM], she put the sutures. She put it loose. After she put it loose, then again some male nurses came and one of them cut the old sutures and put new sutures again. After this they made me sleep outside. At around 1:30, we came back home and sat and gave the baby a bath. After the bath, I was given a bath at 4 o’clock”.*

#### Social and cultural acceptability

Social and cultural acceptability was an aspect of quality care that was central to participant’s discussions of their delivery site preferences and included indicators such as treatment by staff, suitability of the medical interventions and procedures at delivery, ability to enact cultural childbirth practices, and provision of amenities for a “normal delivery”. In all districts, some women reported experiencing socially acceptable treatment and care at government facilities.

*“They saw [*took care*] of me well [at government hospital]. They used to come on time, would come at night and go, they would ask if we were in any kind of pain or anything.”* (New mother, Bagalkot)

However women, primarily in Bellary and Bagalkot and who lived below poverty line, discussed experiences of maltreatment at public institutions but not at the private institutions. Women often discussed experiences of other women at government hospitals that they had heard about.

*“I didn’t go there [to government hospital]… There (the) sisters (nurse/ANM) will beat it seems. So some are afraid of it. That’s why more people won’t go there; they prefer private hospital”.* (New mother, Bellary)

*“There [government hospital], sisters won’t take well care it seems… when madam will check and go they won’t care; they will leave as it is and won’t give tablets also. When I went to (private hospital) they will be available at any time. They check B.P., eyes and baby is breathing or what and will give injection and wake and go if I sleep also. For us it’s good there only, that’s why we prefer to go there”* (Pregnant woman, Bellary).

However, Gulbarga women both above and below the poverty line reported experiencing themselves or hearing about others’ mistreatment to a more severe degree, which is demonstrated by a new mother’s experience at a government facility:

“*They give us injection and then press hard our stomach. Sometimes, they beat us in hospitals*…*They slapped me on face (“kapalakke hodithara”). They beat me on legs (“kaalige hodithara”)… they were scolding (“bayyodu”) me with harsh words taking my parents’ names.”*

Additionally, some women in Bellary and Gulbarga perceived that the poor are ill-treated, particularly at government hospitals. Referring to the government hospital, a scheduled caste new mother who was below-poverty line in Bellary stated that:

“*For poor they will ill treat*”.

Preferences for delivery site were also based on the widespread concern around the cultural expectations for undergoing a “normal delivery”, the components of which included availability of amenities such as hot water and food, mobility after delivery, privacy, and control over the process. The concern over ability to have a normal delivery was expressed by women in all districts. Women in Bagalkot and Bellary compared the advantages for having a normal delivery at home compared to any institutional delivery. A new mother in Bellary whose family took her to deliver at the private hospital stated:

*“Normal delivery is not possible in hospital. They will go for surgery. They don’t give good food. We can’t get up and walk. We should stay in bed for many days… I feel comfort only at home.”* (New mother, Bellary)

The services provided in public institutions were deemed acceptable among some respondents from Bellary and Bagalkot.

*“They (government hospital) look after well… As soon as I became pregnant, from the third month, I started going for checkup at the government hospital. Until the time of delivery, I went to the government hospital only. I never went to the private hospital even one day”.* (New mother, Bagalkot)

However there were also many complaints about public hospitals that didn’t have adequate amenities during delivery.

*“There [at government hospital] we won’t get water. If we tell some other people in surroundings, they will provide water for baby. But we (mothers) don’t get. After coming home only we should take hot water bath. In hospital we won’t get…. Will go to hotel and pay money there and will bring hot water”* (Pregnant woman, Bellary).

*“…in the government hospital if Caesarean happen there will be bleeding and all and they put this for passing urine…at [private facility], they take care of the baby…hot water all facilities…will all be there because it is private…here [government hospital] nothing will be there. New mothers only should do everything but there [private facility] because they take money they do everything (assisting to take care of baby)”* (New mother, Gulbarga)

Preferences were also based on experiences of other people rather than personal experience, as relayed by this new mother from Bagalkot.

*“In Government hospitals they don’t keep the premises neat and tidy, full of dirt only, if someone gets discharged they don’t bother to change the bed covers or cleaning and maintenance are not to be seen”. (*New mother, Bagalkot*)*

The main complaint about the private institutions in terms of social acceptability of treatment was that there was too readily use of C-sections or injections to induce labour even when it could have waited:

“*If you go to the hospital they do Caesarean… when we shout a lot and all they think she has lot of pain and they start one” (Pregnant woman, Gulbarga).*

For many women this was problematic, due their preference for a “normal delivery” compared to an invasive medical procedure.

#### Safety and efficacy

In this study, safety and efficacy in delivery site preferences were discussed in terms of length of labour, level of pain, and perceived safety and comfort of the medical procedures in the antenatal and delivery periods. Many women viewed an institutional birth as more effective for managing pain and heightened safety compared to a home delivery.

“*It is better only in hospital. They provide injection to quicken the delivery. If it is at home, we have to bear the pain for a long time.*” (New mom, Bellary)

*“In home we don’t know anything about it (*giving birth*). If madam* (doctor) *will assist, they will inject and we get enough hope and strength. That’s why we will go there [government hospital]” (*Pregnant woman, Bellary*).*

Yet others did not consider home delivery as unsafe or uncomfortable, and stated that it was good for low-risk or uncomplicated deliveries. For example, a new mother in Bagalkot explained that risk can be assessed during antenatal visits:

*“Baby has grown or not, is it well or not we will come to know about it by scanning”.* (Pregnant woman, Gulbarga)

*“Delivery will be normal so why should I get it done in hospital?”* (New mother, Bagalkot)

Further, caesarean sections at hospitals were seen as less safe and comfortable, and there was a general sense that going to the hospital meant having a Caesarian section.

*“If the delivery is at home, it is best. At home, neighbours or family members will take good care… if it is a normal delivery there is no need to go to the hospital.* (New mother, Bagalkot)

“Where would you like the delivery to take place?”: *“Here in my mother’s house. If you go to the hospital they do Caesarean… when we shout a lot and all they think she has lot of pain and they start one’.* (Pregnant woman, Gulbarga)

## Discussion

Our study sheds light on local understandings and relative importance of health care quality that shaped delivery site preferences of pregnant women and new mothers in three districts of north Karnataka. Women’s perspectives are important because they are the primary service users at the time of delivery and important stakeholders of NRHM interventions. Pregnant and new mothers’ preferences for delivering at home or at private or public hospitals revealed elements of both convergence and multiplicity, and can be summarized in terms of three components of quality of care: a) financial, structural, and geographical *accessibility*, b) social and cultural *acceptability*, and c) *safety and efficacy*. This study is among the first in India to use qualitative methods to explore women’s preferences for place of delivery and the factors influencing their perspectives. There have been few mixed-methods or qualitative studies that have assessed the quality of care or reasons for delivery site preferences at public versus private institutions in India [10,15-17]. Instead, most previous studies on birthing location have been quantitative analyses of large-scale National Family Health Surveys or smaller-scale surveys that have assessed factors associated with different delivery sites [18-23].

Participants in our study gave their views on the three delivery sites: home, private or public (government) institutions. Government hospitals were seen as having relevant and effective medical treatment and services in some cases, but many respondents also discussed the lack of quality components such as basic amenities (e.g. water), cleanliness, and control over the situation. Women did not highlight the lower cost of government hospitals as a particular reason for preferring those facilities, but did mention the drawback of costs that could be incurred at government hospitals. Receipt of incentives was variable, and access to government hospitals by ambulance with the help of the ASHAs was sometimes but not always possible. Social acceptability was a major point of discussion among study participants. Treatment by staff at government hospitals was inconsistent and in some cases, particularly in Gulbarga, there were experiences of maltreatment. This inconsistency in quality of care at government hospitals was echoed in a study completed across rural India [20]. Similar experiences of poor treatment were found in the slums of New Delhi, where 15% of women reported being shouted at or slapped during labour at government institutions, compared to 3% during private hospital deliveries [21].

In contrast, private hospitals were seen in a more favourable light, with more consistently positive views. Treatment by staff and the services provided were seen to be acceptable. However, some women stated that C-sections were more common at private hospitals, and other commonly occurring medical procedures were too invasive and potentially unsafe, thus they were viewed with wariness. This supports the findings by Hulton and colleagues’ study on the quality of hospitals in a New Delhi slum [21]. They found that women delivering at government hospitals reported mixed treatment while those delivering at private hospitals generally reported more positive treatment. In their study they also found an over-use of medical procedures at private hospitals, which they linked to the profits that can be made by doing C-sections: “the costing of clinical interventions by private providers provides a financial incentive which may compromise clinical judgment. The reverse is true for the public sector… Neither of these situations is optimal in terms of supporting high quality institutional maternity care” [21]. Financial access was the most negative characteristic of private care according to the women, which led to the perception that private hospitals are exclusively for wealthier families and therefore associated with some inequity. Nonetheless, some saw the high cost as a marker of quality, which may have combined with the positive actual and perceived impressions on the quality of care at private institutions. The wide preference for private hospitals in India supports Thind and colleagues’ finding that when the mothers were the primary decision-maker, the majority preferred the private hospital delivery in Maharashtra state [23].

The views around home delivery were also mixed and not simply based on a lack of education or poverty level of the users [24]. While home delivery was generally becoming less favourable among the younger generation, it remains a preferable option for non-complicated deliveries by many women and was sometimes determined at antenatal care visits. Thus, the socio-cultural acceptability of having a “normal delivery” at home, often attended by a traditional birth attendant or occasionally by a doctor, made it preferable to delivering at hospitals. These were similar to the reasons for home birth found elsewhere [24]. Further, access was not an issue for home delivery as there were no service costs associated and no need to travel externally. Yet there were some women in all districts who stated that home delivery was more risky and painful in general and thus less safe or preferable compared to hospital delivery.

An analysis of the components of quality of care in relation to each delivery site in respondents’ accounts revealed some key themes with implications for MNCH interventions in the region. The first theme is that both personal and others’ experiences of quality of care influenced women’s preferences for a certain delivery site. Negative experiences, whether experienced personally or by others, had a significant impact on the perceptions women held of a given delivery site and thereby reinforced their preferences. Inconsistent experiences at government hospitals compared to more consistently positive experiences and views of private hospitals led to a preference for the latter. This is consistent with Sidney and colleagues’ finding that 30% of 418 new mothers in Ujjain district of Madhya Pradesh used private facilities because of their good reputation or past experiences [24]. The perception that home delivery was becoming generally less favourable, but appropriate when there was an uncomplicated delivery, was also important in guiding their preferences. This is also supported by Sidney and colleagues’ findings that delivering at home was related to whether the mother “felt previous deliveries were easy so there was no need” [24]. Thus, it is important to improve women’s experiences at government hospitals in areas deemed important to them so as to improve perceptions and therefore facilitate increased uptake of government facilities for infant delivery.

The second theme centres on the continued importance of access issues and the need for more consistent outreach, better ambulance services, and more efficient receipt of financial or other incentives. Not all women were able to travel to the hospitals by ambulance because of limited availability, which was also found in Sidney and colleagues’ study [24]. Nor did participants’ always know about or receive incentives. Thus, the lesser costs associated with government hospitals and the possibility of incentives for delivering at government hospitals did not diminish preference for and perceptions of better care and outcomes at private hospitals. The high costs of the private hospital concerned all women regardless of their positioning in relation to the poverty line. This contrasts with the finding in other studies that being above the poverty line correlated with institutional and especially private delivery [19,20,23,25]. Our finding may be explained in part by Sidney and colleagues’ observation that the positive reputation of private institutions was more important than socio-economic status with respect to delivery site choices, and that while some rural women may be above the poverty line, they may still be relatively poor compared to those in urban areas of India [24]. The importance placed on quality in terms of acceptability and safety, which was generally seen as higher at private hospitals, further fuelled the concern about access to private deliveries. At the same time, greater costs also were sometimes seen as a marker of quality of care at the private hospitals. This suggests that solely incentivizing government hospital delivery with a cash transfer under the JSY scheme, without addressing the other issues women consider in deciding on delivery site, will continue to lead women and possibly other decision-makers in their families to desire private deliveries. This has been found previously [15,21,22,24,25]. Improving equity through the provision of schemes to those below poverty and scheduled tribe and caste has been a challenge according to other studies that show usage of the JSY scheme to be below 50% among these groups in other Indian states [26]. However, the scaling up of accessible care, particularly to hard-to-reach populations, remains important.

Finally, despite ongoing access issues particularly for the most marginalized, the increasing numbers of institutional deliveries in Karnataka on average suggests that particular attention must be given to social and cultural acceptability as well as safety so as to improve perceptions and experiences when delivering at health facilities [7]. Many studies have focussed their recommendations on supply-side issues including training more physicians and supplying more equipment and facilities in rural areas [17,22,25,27,28]. As shown in the results of our study, the focus of NRHM interventions centers heavily on improving financial and geographical access through incentives and outreach workers to connect women to the facility. Yet in addition to this, women’s priorities and definitions of quality care in this study were directly linked to the quality of the experience at the time of delivery, including social acceptability and safety. We found that the respondents’ preferences were greatly influenced by the ability to undergo a “normal delivery” with culturally desirable and relevant amenities such as hot water, warmth and food, and with more control over the birth process. Another important factor of quality that shaped their preferences was the experience of maltreatment, particularly towards poorer women and at government institutions. This led many to favour private institutions or home delivery rather than government facilities. Previous studies have not stressed the importance of social and cultural acceptability in these terms [15,27], and few have stressed the need for improved treatment of lower caste or below-poverty line women [19], despite the explicit efforts and mission of the NRHM to improve equity through improving access to institutional delivery [14]. In their research on health-seeking behaviour, Haussman-Muela and colleagues have emphasized the need to consider the acceptability of health personnel-client dynamics, citing studies that found factors such as poor explanations on treatment, rudeness, or blaming-the-victim mentalities as potential barriers for health care. At the same time, they stress the importance of contextualizing these behaviours, for example considering the salaries, work conditions and institutional culture that may reproduce inequitable power relations and related abuse within health centres [29]. Thus, quality of care, especially as government institutions are made more accessible, must be improved in line with women’s expectations, particularly in terms of staff treatment, provision of acceptable and safe care, and where possible, conducting “normal” deliveries with appropriate amenities.

This study has limitations. While the sample size was quite large for a qualitative study and we attempted to maximize variation in terms of relevant socio-demographic characteristics, it was not a representative sample and therefore the findings cannot be generalized to the whole population. However, with a qualitative design, the aim is to gather in-depth information to explore the variation in views rather than to determine generalizable relationships between factors. Though we attempted to compare socio-economic groups based on caste and whether respondents were above or below the poverty line, we found that respondents in all categories saw themselves as relatively poor, and though this is a relevant finding we cannot use this to make broad conclusions on how differences in socio-economic status impact delivery preferences. We developed categories inductively from the data and have gone over the interpretations of the results with stakeholders in India, however it is possible that researcher and stakeholder bias has some influence on the interpretation of the findings. Notwithstanding these limitations the study has numerous strengths. First, we recruited a large sample of women from various backgrounds and were able to conduct interviews in their most familiar language. Second, the study is grounded in the activities and experiences of those in the local environment. Partners and stakeholders represent Indian researchers, policy makers, decision-makers, and health care providers. In consultation with local communities the topic was deemed relevant and important. Third, local research assistants underwent thorough training and ongoing feedback during data collection. Finally, local women, policy-makers, decision-makers and health care providers participated in the analysis and interpretation of the findings.

## Conclusion

The focus of efforts to address deficiencies in MNCH, in both the literature and in national strategies, has largely been on improving access on the demand side, and on providing more trained doctors, facilities, and equipment on the supply side [14,22,25]. However, local definitions of quality of care among women who use services for delivering their children place great emphasis on treatment by staff and on the provision of socio-culturally acceptable and safe facilities and procedures at delivery as well. Based on these priorities, women in this study preferred private hospitals to a large extent. Yet this preference for private delivery conflicted with their concern over accessibility, including the high costs associated with them and the limits of incentive programs to use them. Overall, these findings suggest that improvements in availability, accessibility, quality and ultimately utilization of MNCH services are all interrelated, and will be better achieved in concert with one other rather than independently.
